# Periostin: its role in asthma and its potential as a diagnostic or therapeutic target

**DOI:** 10.1186/s12931-015-0218-2

**Published:** 2015-05-17

**Authors:** Wei Li, Peng Gao, Yue Zhi, Wei Xu, Yanfeng Wu, Jinzhi Yin, Jie Zhang

**Affiliations:** Department of Respiratory and Critical Care Medicine, the Second Affiliated Hospital of Jilin University, Changchun, Jilin 130041 China

**Keywords:** Periostin, Inflammation, Eosinophils, Airway remodeling, Asthma

## Abstract

Accumulating evidence shows that periostin, a matricellular protein, is involved in many fundamental biological processes such as cell proliferation, cell invasion, and angiogenesis. Changes in periostin expression are commonly detected in various cancers and pre-cancerous conditions, and periostin may be involved in regulating a diverse set of cancer cell activities that contribute to tumorigenesis, cancer progression, and metastasis. Periostin has also been shown to be involved in many aspects of allergic inflammation, such as eosinophil recruitment, airway remodeling, development of a Th2 phenotype, and increased expression of inflammatory mediators. In an *in vivo* model, bronchoalveolar lavage (BAL) fluid obtained from ovalbumin-challenged mice was found to contain significantly higher levels of periostin compared to BAL samples from control mice. To date, the molecular mechanisms involving periostin in relation to asthma in humans have not been fully elucidated. This review will focus on what is known about periostin and its role in the pathophysiological mechanisms that mediate asthma in order to evaluate the potential for periostin to serve as a biomarker and therapeutic target for the detection and treatment of asthma, respectively.

## Introduction

Periostin, also referred to as osteoblast-specific factor2, was first described in 1993 and was named based on its expression in the periodontal ligament and periosteum of adult mice [1]. Subsequent studies demonstrated that periostin is ubiquitously expressed in a wide variety of normal adult tissues and fetal tissues, including embryonic periosteum, periodontal ligament, placenta, cardiac valve, adrenal gland, lung, thyroid tissues [2].

Periostin is a 90-kDa member of the fasciclin-containing protein family and is encoded by the *POSTN* gene in humans (GenBank accession no., D13664). Periostin is a matricellular protein that mediates cell activation by binding to receptors present on the cell surface [3–5]. Periostin is a secreted protein that shares structural homology with the axon guidance protein, FAS1, in insects [6]. In addition, periostin is highly homologous with transforming growth factor (TGF)-β-induced protein, βig-h3,which promotes cell adhesion, the development of cardiac valves [7], and the spreading of fibroblast [8], epithelial [9], and ovarian cells [10]. Periostin is expressed at higher levels in patients affected by conditions that are associated with increased cell division, cell turnover, cell invasion, and angiogenesis [11].

More recently, periostin has been recognized as having important roles in the development of bone, tooth, and heart valves, as well as during the healing process after myocardial infarction and in the development of various tumors [12]. Furthermore, periostin has been implicated in atopic conditions such as dermatitis [13] and rhinitis/rhinosinusitis [14]. In allergic skin inflammations, periostin induction after an initial injury contributes to the establishment of sustained chronic inflammation and tissue remodeling [15]. Chronic rhinosinusitis inflammation is mediated by periostin and osteopontin, and these proteins induce a proliferative response within the extracellular matrix (ECM) framework which leads to large scale remodeling of sinus histopathology [14]. Increased expression of periostin in tissues has also been associated with several inflammatory conditions that have been investigated in the fields of eosinophilia (e.g., otitis media, eosinophilic esophagitis), ophthalmology (e.g., proliferative diabetic retinopathy), hematology (e.g., bone marrow fibrosis),and fibrotic remodeling (e.g., immunoglobulin (Ig)G4-related sclerosing sialadenitis and scleroderma) [5].

The role of periostin in asthma and type 2 inflammatory responses is an area of active research. Recently, Sehra et al. and Gordon et al. demonstrated that periostin protects mice from allergic airway inflammation, whereas Blanchard et al. showed that periostin accelerates allergen-induced eosinophil recruitment in the lung and esophagus [16–18]. A similar protocol using intranasal administration of *Aspergillus fumigatus* (*A. fumigatus*) led to different outcomes, thereby suggesting that the role of periostin in allergic airway inflammation remains unclear [15].

A newly proposed approach to asthma classification is based on “endotypes” that represent specific cellular patterns. In combination with clinical characteristics, it is proposed that these endotypes can establish patient subgroups. Correspondingly, an analysis of induced sputum samples allowed inflammatory phenotypes to be determined according to granulocytic composition, namely eosinophilic, neutrophilic, mixed granulocytic, or paucigranulocytic [19].

In recent decades, the number of studies involving periostin has been rapidly growing. This is partly related to the wide range of functions observed for periostin. Periostin has been found to be related to the physiopathology of multiple diseases such as: ankylosing spondylitis (AS) [20], idiopathic interstitial pneumonias (IIPs) [21], idiopathic pulmonary fibrosis (IPF) [22], renal inflammation and fibrosis [23], heart aging [12], cancer [24, 25], and allergic diseases (Table 1). Periostin is involved in many aspects of asthma as well, including eosinophil recruitment [12], airway remodeling, development of a Th2 phenotype, and contributes to the increased expression of inflammatory mediators [26, 27]. This review will focus on what is known about periostin and its role in the pathophysiological mechanisms that mediate asthma.

**Table 1:** Serum levels of periostin in allergic diseases

Disease	Assays to detect periostin	Level in blood (pg/ml)	Comments
Allergic fungal rhinosinusitis (AFRS)	IF and semi-quantitative RT-PCR	Periostin was significantly elevated in AFRS sinus tissue compared to CRSsNP and controls by IF (p < 0.001) and PCR (p = 0.011).	Periostin levels positively correlate with radiologic disease severity scores. Increased levels of periostin in AFRS are possibly tied to its intense eosinophilic inflammatory etiology [39].
Chronic rhinosinusitis (CRS)	IHC	Higher expression of POSTN was observed in the CRS group compared to controls (FC = 4.89, pFDR = 0.0006), which was also verified by IHC. After ESS, POSTN expression in the CRS group decreased (FC = −3.074, pFDR = 0.0044), and no longer differed from the controls (FC = 1.56, pFDR = 0.3).	Reduced gene expression of periostin following resolution of disease suggests that POSTN may represent a pathogenesis indicator or biomarker of CRS disease activity and responsiveness to treatment [74].
Atopic Dermatitis (AD)	ELISA	Serum levels of periostin in adult patients with AD (n = 257), patients with PV as a disease control (n = 66), and 25 healthy controls were assayed. Serum periostin was significantly higher in patients with AD than the other two groups. Periostin levels positively correlated with disease severity, TARC levels, LDH levels, and eosinophil counts, but not with IgE levels.	Periostin may play a critical role in disease severity and chronic status in the pathogenesis of AD [33].
Aspirin-exacerbated respiratory disease (AERD)	ELISA	Serum periostin levels were significantly higher in patients with AERD vs. ATA, in patients with severe asthma vs. nonsevere asthma, and in patients with eosinophilic asthma vs. noneosinophilic asthma (P = 0.005, P = 0.02, and P = 0.001, respectively).	Serum periostin levels are significantly elevated in AERD patients and are associated with AERD phenotype and disease severity [71].
Asthma	ELISA	Serum periostin levels were significantly higher in asthmatic patients with evidence of eosinophilic airway inflammation relative to those with minimal eosinophilic airway inflammation.	Periostin is a systemic biomarker of airway eosinophilia in asthmatic patients and has the potential to be used for patient selection for emerging asthma therapeutics that target Th2 inflammation [36].

*IF* immunofluorescence, *IHC* immunohistochemistry, *RT-PCR* reverse-transcription polymerase chain reaction, *CRSsNP* chronic rhinosinusitis without nasal polyps, *FC* fold-change, *pFDR* positive false discovery rate, *ESS* endoscopic sinus surgery, *ELISA* enzyme-linked immunosorbent assay, *PV* psoriasis vulgaris, *TARC* thymus and activation-regulated chemokine, *LDH* lactate dehydrogenase, *ATA* aspirin tolerant asthma

### Periostin in inflammation

Asthma is a chronic inflammatory respiratory disease that is commonly characterized by airway inflammation, airway hyperresponsiveness (AHR), and/or reversible airway obstruction. To date, there are treatments available that target eosinophilic inflammation in asthma, and these have been able to reduce asthma exacerbations in some cases [28]. However, the inflammatory mechanisms leading to asthma symptoms and AHR in the absence of sputum eosinophilia are poorly understood. Periostin is potentially relevant in the pathogenesis of asthma-associated inflammation and its phenotypes [29, 30].

### Periostin expression in the inflammatory setting

A variety of tissues and cell types express periostin under basal conditions, including epithelial cells, fibroblasts, and eosinophils [9, 31, 32]. However, the pattern of expression can be modulated in response to inflammation. For example, in mice exposed to house dust mites (HDMs), periostin expression was found to increase in the airway epithelium, subepithelium, smooth muscle, and inflammatory cells, while mice that received an injection of OC-20 (a neutralizing antibody to periostin) exhibited reduced airway responsiveness following exposure to HDMs [26]. HDM exposure also increased airway responsiveness in *Postn*^+/+^ mice and not in *Postn*^−/−^mice [26]. For patients with atopic dermatitis (AD), serum levels of periostin were significantly higher than in patients with psoriasis vulgaris (PV) and healthy controls [33]. Moreover, in work by Covone et al. and Kanemitsu et al., periostin levels were found to positively correlate with juvenile idiopathic arthritis and asthma disease severity, respectively [34, 35]. In addition, periostin levels positively correlated with thymus and activ ation-regulated chemokine (TARC) levels, lactate dehydrogenase (LDH) levels, and eosinophil counts, yet not with IgE levels [33]. In asthma patients, gene expression of periostin in sputum cells and elevated serum levels of periostin were detected by PCR and ELISA, respectively [36, 37]. Periostin has also been shown to promote inflammation in experimental models of allergic disease, including models of HDM-induced atopic dermatitis, ovalbumin (OVA)-induced allergic rhinitis, and *A. fumigatus*-induced eosinophilices ophagitis [26].

In vitro assays have confirmed that expression of POSTN and TWIST1 are elevated in fibroblast-like synoviocytes (FLSs) of rheumatoid arthritis patients (RA-FLSs) and are crucial for the migration and invasion of FLSs stimulated with interleukin (IL)-1β [38]. Moreover, regulation of FLS migration by *POSTN* and TWIST1 was confirmed in an *in vivo* animal model of skin inflammation [38]. These findings are consistent with the up-regulation of periostin that is observed with immune activation, and with the roles of dietary fat and IL-1β in innate immune activation [28]. Furthermore, epithelial cells and fibroblasts in vitro produce large amounts of periostin, and these are the major cell types that contain periostin [32]. Increased levels of periostin have also been detected in relation to neutrophils, eosinophils, mast cells, monocytes, and lymphocytes.

### Regulation of leukocyte trafficking and activation

In studies of IPF, inflammation has been found to precede the onset of fibrosis. Moreover, when IPF was induced in wildtype and periostin-deficient mice with administration of bleomycin (BLM), periostin-dependent infiltration of neutrophils and macrophage were observed, while accumulation of periostin was not detected [12]. These results suggest that the basal concentration of periostin present in lung tissue is sufficient for an acute response, and it is possible that accumulated levels of periostin may enhance or sustain IPF-associated inflammation [12]. Given that previous studies have demonstrated that periostin also plays a critical role in the trafficking, activation, and cytokine release of leukocytes [39, 40], these findings suggest that periostin contributes to esophagitis, and periostin may directly regulate leukocyte (eosinophil) accumulation in T helper type 2-associated mucosal inflammation in both mice and humans [18].

### Eosinophils

**Fig. 1:**
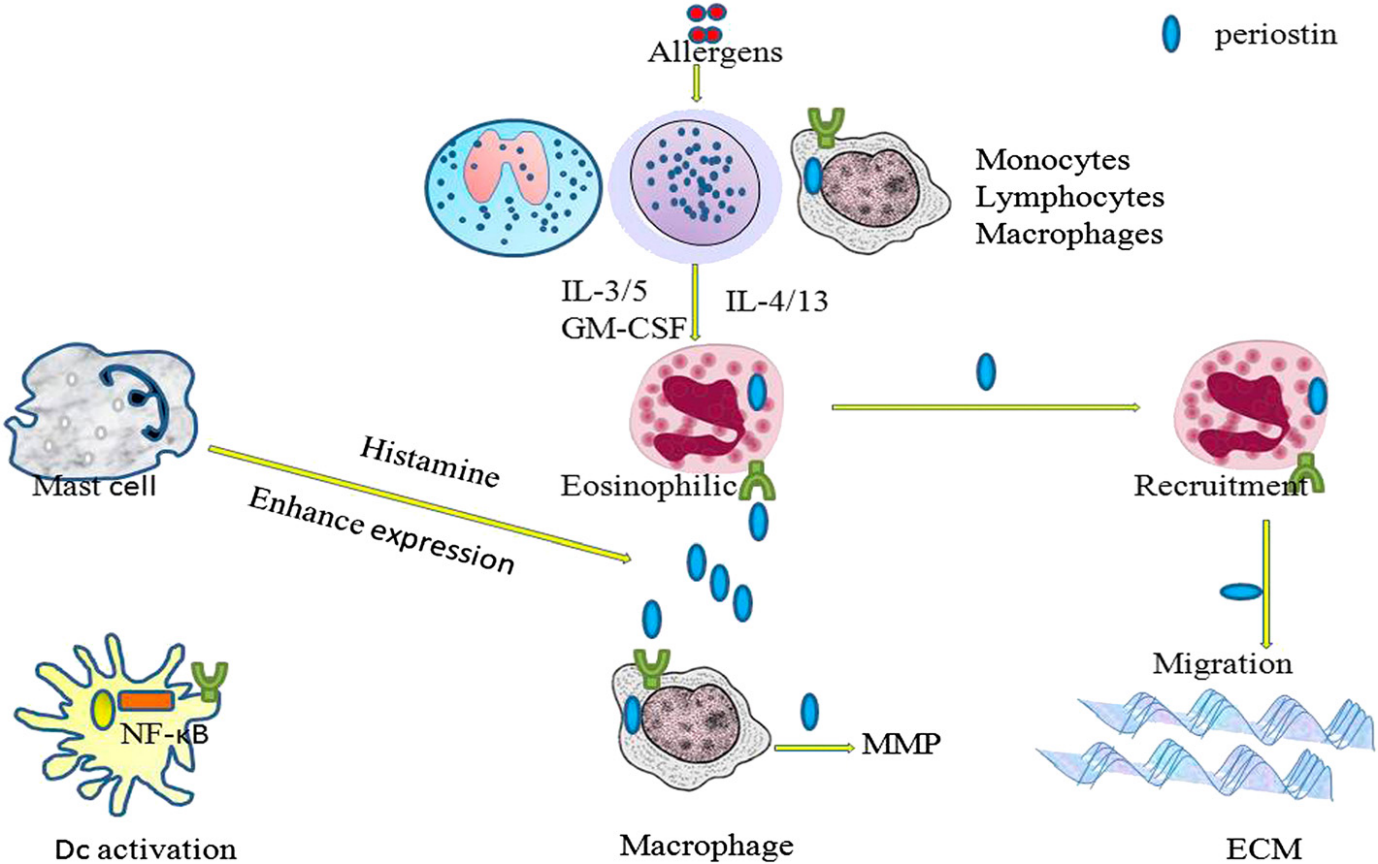
Periostin is involved in the pathogenic process of eosinophils and Th2-type asthma. Briefly, allergens induce the secretion of IL-4 and IL-13 from certain immune cells, thereby stimulating eosinophils cells to produce periostin. Periostin, in turn through an autocrine pathway (indicated by the green colored receptors), acts on eosinophils to stimulate the adhesion of purified human blood eosinophils, while also enhancing their recruitment to an asthmatic airway. IL-5, IL-3, or GM-CSF can also stimulate the adhesion of purified human blood eosinophils, thereby leading to a vicious cycle. Macrophage produce periostin, and this can enhance the secretion of matrix metalloproteinases (MMPs). Furthermore, in the presence of histamine, expression of periostin by other cells is enhanced

**Fig. 2:**
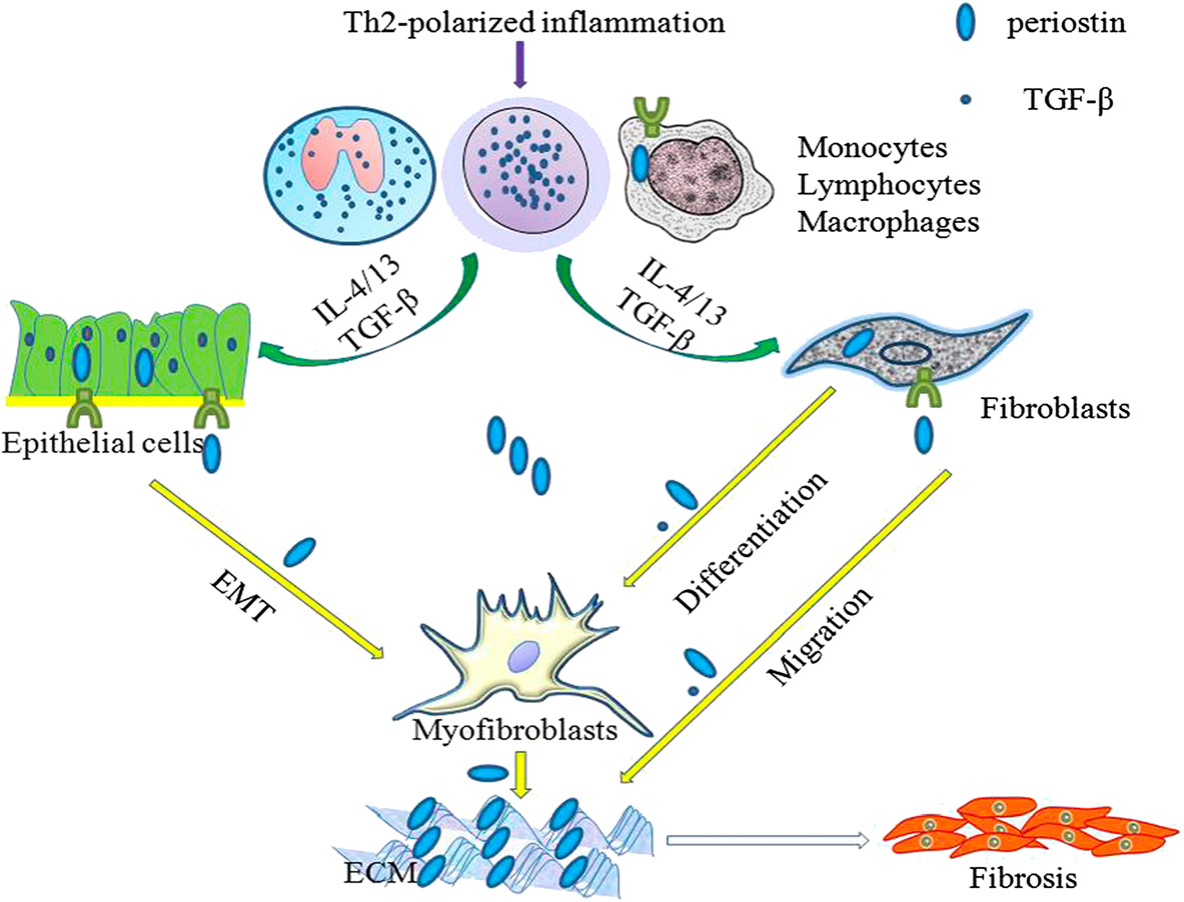
The role of periostin inthe pathogenic process of subepithelial fibrosis. Briefly, Th2-polarized inflammation leads to the recruitment of various immune cells and these secrete IL-4 and IL-13 in combination with TGF-β to stimulate fibroblasts and epithelial cells to produce periostin. Periostin, in turn through an autocrine pathway (indicated with green colored receptors), acts on fibroblasts and epithelial cells, thereby leading to a vicious cycle. Additional functions of periostin include: the promotion of fibroblast differentiation into myofibroblasts, the induction of fibroblast migration, and as a co-factor of TGF-β, periostin promotes ECM production and the differentiation of myofibroblasts. Periostin further enhances fibrosis by binding to other ECM proteins (e.g., collagen I, fibronectin, and tenascin-C) and by inducing collagen fibrillogenesis. Adapted from [27]

An increasing number of studies have confirmed that plasma levels of periostin are higher in patients with eosinophilic asthma [41, 42]. In mice, the numbers of eosinophils tend to be lower in periostin-deficient mice compared with wild-type mice, although the down-regulation is not statistically significant [12]. A functional role for periostin in eliciting esophageal eosinophilia has been demonstrated using periostin-null mice, where allergen-induced recruitment of eosinophils to the lung and esophagus have been found to undergo a 66 and 72 % decrease, respectively [18]. Periostin-null mice also respond to lung challenges with significantly lower numbers of eosinophils in the lung and higher numbers of eosinophils in blood, and they exhibit reduced inflammation in an allergic skin inflammation model [5, 43]. To explain why periostin levels increase with asthma, it has been hypothesized that eosinophils secrete periostin [31]. However, other researchers have proposed that eosinophilic asthma derives from the production of periostin mainly by airway epithelial cells and fibroblasts [44–46]. However, if eosinophils can secrete periostin, what regulates this production? Periostin has been shown to act on eosinophils, thereby enhancing their recruitment to lesions, and acts on airway epithelial cells to induce TGF-β activation, as part of the pathogenesis of bronchial asthma [12]. In Figs. 1 and 2, the relationship between periostin and airway epithelial cells and fibroblasts is illustrated in relation to Th2-type asthma and subepithelial fibrosis, respectively [6, 9, 31, 32, 44–48]. In both mouse and human studies, periostin appears to promote eosinophil recruitment, and to enhance the specific adhesion of blood eosinophils that are stimulated by IL-5 or the related cytokines, IL-3 and granulocyte-macrophage colony-stimulating factor (GM-CSF) [43]. Correspondingly, the intranasal inhalation of allergens from *A. fumigatus* in a recent study of periostin-deficient mice demonstrated that fewer eosinophils were recruited to the lung, concomitant with an increase in blood eosinophilia [16]. Moreover, no significant differences in mucus production were detected. Additional studies demonstrated that periostin, as a ligand for the integrins, α_v_β_3_, α_v_β_5_, α_4_β_6_, and α_M_β_2_ (CD11b), was able to attract eosinophils via CD11b, and increased the adhesion of eosinophils to fibronectin [26]. In vitro studies further indicated that α_4_β_1_ and α_M_β_2_ are the principal integrins mediating eosinophil adhesion, particularly in relation to vascular cell adhesion molecule-1 and the novel α_M_β_2_ ligand, periostin [49]. *In vivo*, blood eosinophils express β_1_ integrins that have been characterized by intermediate activity and intermediate conformations in studies performed using monoclonal antibodies (mAbs) N29 and KIM-127, respectively, potentially due to eosinophil binding of P-selectin on the surface of activated platelets and exposure to low concentrations of IL-5, respectively [49]. In contrast, airway eosinophils recovered by bronchoalveolar lavage (BAL) after segmental antigen challenge express high-activity β_1_ and high-activity α_M_β_2_ integrins that do not require IL-5 [49].

Intense eosinophilic infiltration is closely related with an elevated production of cytokines and chemokines such as IL-5 and eotaxin [50]. For example, in an in vitro model, periostin was adsorbed to polystyrene and adhesion of purified human blood eosinophils stimulated by IL-5, IL-3, or GM-CSF was supported. In contrast, the adhesion of eosinophils treated with IL-4 or IL-13 was not supported [43]. Furthermore, the degree of adhesion achieved was found to depend on the concentration of periostin present during the coating and activation by cytokines during the adhesion assay. Interestingly, both full-length periostin and a spliced version of periostin that lacks the C-terminal exons, 17, 18, 19, and 21, have been found to support adhesion. However, in the presence of a monoclonal antibody raised against α_M_ or β_2_ integrin subunits, versus antibodies raised against other eosinophil integrin subunits, only the former inhibited adhesion. Adsorbed periostin was also observed to support α_M_β_2_-dependent random motility of IL-5-stimulated eosinophils, with optimal movement observed at an intermediate coating concentration. In the presence of IL-5, eosinophils adhere to periostin and form punctate structures that express filamentous actin, gelsolin, and phosphotyrosine. This profile is consistent with that observed for podosomes, which represent highly dynamic adhesive contacts that are distinct from classical focal adhesions. In work by Johansson et al., α_M_β_2_ (CD11b/CD18, Mac-1) was identified as an adhesive and promigratory periostin receptor that is expressed by cytokine-stimulated eosinophils. Thus, periostin may function as a haptotactic stimulus to guide eosinophils to asthmatic airways where high levels of periostin are present [43]. Deposition of periostin in airways as a result of Th2 immunity may also promote eosinophil recruitment and serve as a guide for eosinophil migration in the ECM of asthmatic airways. Correspondingly, it is possible that a periostin gradient may complement the gradients of the chemotactic agents that eosinophils are exposed to, thereby leading to the arrest of cells at a critical density of periostin. Furthermore, interactions with periostin may also affect eosinophil survival and mediator release, and this may provide an opportunity to modulate allergic inflammation and airway remodeling [43]. IL-4, IL-13, and TGF-β are major triggers of periostin production [51]. Thus, while IL-5, IL-3, and GM-CSF have the capacity to stimulate the adhesion of purified human blood eosinophils, IL-4 and IL-13 may trigger periostin production. Further experiments are needed to investigate these possibilities, although an autocrine loop involving periostin and eosinophils could lead to a vicious cycle of signaling.

Cell classification, especially in relation to levels of periostin, may have important significance for cases of eosinophil asthma. In a study of periostin concentration in sputum by Bobolea et al., periostin concentration and numbers of eosinophils were found to be closely related [29]. However, in another study, serum levels of periostin did not distinguish eosinophilic airway inflammation from non-eosinophilic airway inflammation [52]. Therefore, additional experiments need to be performed to investigate whether periosteum has a differential role in various asthma cell types.

### Macrophage

*In vivo*, the infiltration of macrophage in BAL fluid is significantly reduced in periostin-deficient mice compared with wild-type mice [12]. Thus, periostin may serve as a chemoattractant and adhesion factor to affect the trafficking of macrophage. In vitro studies have showed that M2-polarized macrophage significantly increases mRNA levels of periostin, and this induction may promote retinal neovascularization and fibrosis [53, 54]. *In vivo*, immunohistochemistry assays have detected weak expression of periostin in reactive astrocytes in the periinfarct region and in microglia/macrophage in infarct regions on days 3 and 7 post-infarct, respectively [55]. Furthermore, both in vitro and *ex vivo* studies have revealed that periostin promotes tube formation and the mobilization of endothelial cells (ECs), and prominently increases matrix metalloproteinase secretion from cultured valvular interstitial cells, ECs, and macrophage in a cell type-specific manner [56]. Thus, macrophage have many important roles in processes such as cell proliferation and cell remodeling, in airway hyperresponsiveness and corticosteroid resistance, and in asthma exacerbations and airway remodeling [57]. It is also hypothesized that pulmonary macrophage produce periostin through an autocrine function of periostin and this may contribute to the pathogenesis of asthma. Correspondingly, investigations of the mechanisms that mediate the function and regulation of macrophage are ongoing and are of great interest in the field of asthma research, even in studies of asthmatic inflammation. Furthermore, accumulating evidence suggests that cell type is a key consideration for the treatment of asthma, and it has extraordinary significance.

### Neutrophils

Periostin provides a cell-binding matrix which can facilitate the infiltration of inflammatory cells in the airways [26]. It has been observed that neutrophils are the first cells recruited to the site of an allergic reaction, and thus, neutrophils may influence clinical presentation. Correspondingly, neutrophils have been linked to the development of severe chronic asthma, as well as sudden severe attacks of asthma [58].

Inflammation induces the expression of TGF-β and/or IL-4 and IL-13 in macrophage and neutrophils, leading to the expression of periostin and other ECM molecules such as fibronectin and tenascin-C in fibroblasts [59]. Periostin can attract neutrophils and other inflammatory cells to the airway, similar to eosinophils. However, neutrophils can also promote the release of periostin through the secretion of cytokines. When Bentley et al. examined the effects of periostin deficiency on inflammatory cells in BAL fluid and in lung tissue, exposure to HDMs increased neutrophil, macrophage/monocyte, lymphocyte, and eosinophil numbers in BAL fluid samples from *Postn*^+/+^ mice, while *Postn*^−/−^ mice exposed to HDMs had fewer lung Grl^+^ neutrophils, TCR-β^+^ T cells, and Grl^+^ and Siglec-F^+^ eosinophils [26]. Immunohistochemical stainings also showed that HDM exposure increased periostin expression in the airway epithelium, subepithelium, and inflammatory cell infiltrates, while periostin expression was absent in *Postn*^−/−^ mice [26]. Uchida et al. previously demonstrated that periostin is able to synergistically induce the production of several chemokines and cytokines in response to TNF-α and IL-1β [12]. Similarly, the present findings suggest that both TNF-α and periostin are required for the maximal production of chemokines. However, in the former study [12], it was also reported that in the absence of periostin, Ccl4 and IL-1 were not induced by TNF-α, and IL-1 signaling was required, and was sufficient on its own, to mediate BLM-induced pulmonary inflammation and fibrosis. It was further demonstrated that activation of extracellular signal-regulated kinases 1 and 2 (ERK1/2), as well as phosphatidylinositol 3-kinase (PI3K), is important for TNF-α-mediated activation of NF-κB and activator protein-1 [12]. Correspondingly, the authors hypothesized that periostin may enhance TNF-α signaling via ERK1/2 and PI3K. Accumulating evidence also suggests that IL-1β may play an important role in the pathogenesis of asthma [60]. It is possible that periostin can further lead to a malignant role for asthma since periostin can enhance the production of IL-1. In a study by Hayashi et al., nasal administration of OVA and IL-18 was found to act on memory type T helper (Th)-1 cells to induce AHR and inflammation characterized by peribronchial infiltration by neutrophils and eosinophils [61]. These Th1 cells were also found to secrete several types of cytokines, including IFN-γ and the bronchogenic cytokine, IL-13, when stimulated with antigen (Ag) and IL-18. Neutralization of IL-13 during the Ag plus IL-18 challenges inhibited eosinophilic infiltration, lung fibrosis, and periostin deposition; while neutralization of IFN-γ during the Ag plus IL-18 challenges inhibited neutrophilic infiltration and AHR [61]. Thus, periostin appears to coordinate with both eosinophils and neutrophils, although the mechanisms involved remain to be fully characterized. It is possible that periostin may serve as a marker of cell classification and/or a therapeutic target for the treatment of asthma, and additional studies are needed to investigate these possibilities.

### Other cells

#### Dendritic cells (DCs)

In a very recent study, Bentley et al. reported that lower levels of IgE were detected following the treatment of periostin-depleted mice with OC-20. Correspondingly, the authors hypothesized that interactions between CD11b^+^ DCs and periostin facilitate the activation of T cells and Th2 differentiation [26]. CD11b^high^ DCs were also found to mediate CD4^+^ Th2 responses to OVA and HDMs [26]. In the same study, DCs isolated from *Postn*^−/−^ mice exposed to HDMs did not express CD86 [62]. Binding of CD86 by CD28 normally provides CD4^+^ T cells with a costimulatory signal that decreases their activation threshold and enhances their activation [26]. When splenic T cells were incubated with the same DCs isolated from *Postn*^−/−^ mice, or with DCs treated with OC-20, deficient HDM-induced IL-13 responses were observed compared with T cells incubated with wild-type or IgM-treated DCs. Furthermore, bone marrow-derived DCs from wildtype mice were sufficient to promote allergic responses in periostin knockout mice exposed to HDMs. Lastly, in vitro, periostin has been shown to activate nuclear factor (NF)-κB in keratinocytes, consistent with a role for NF-kB in DC activation [26]. Taken together, these results suggest that periostin is required for maximal DC activation.

#### Goblet cells

In *Postn*^−/−^ lungs versus *Postn*^+/+^ lungs, increased airway resistance and significantly enhanced mucus production by goblet cells is observed, concomitant with increased expression of Gob5, a putative calcium-activated chloride channel involved in the regulation of mucus production, and Muc5ac [16]. Correspondingly, in wild-type mice, periostin has been found to inhibit the expression of Gob5 in primary murine airway epithelial cells [16]. Periostin may also bind integrins α4 and β1/2, which have roles in mediating asthma, thereby triggering intracellular signaling pathways that repress mucus-inducing transcription factors such as NF-κB, Sp1, and AP-1 [63]. Data reported by Sehra et al. demonstrate that in the absence of periostin, increased differentiation of epithelial cells into mucus-producing goblet cells occurs, thereby suggesting that periostin contributes to the homeostasis of goblet cell metaplasia (GCM) during allergic inflammation [16]. Periostin can also reduce the symptoms associated with asthma.

#### Mast cells

Human mast cells express functional receptors for IL-3, IL-5, and GM-CSF. In the presence of stem cell factor (SCF), IL-3 has been shown to enhance mast cell growth by decreasing levels of mast cell apoptosis [64]. Correspondingly, loss of periostin appears to enhance the formation of polyp-like lesions and the infiltration of mast cells in a mouse model of eosinophilic rhinosinusitis with nasal polyps [65]. Furthermore, histamine has been found to directly induce periostin expression in a dose-dependent manner in wild-type fibroblasts, and induction of periostin and collagen by histamine involves activation of the H1 receptor-mediated ERK1/2 pathway [66].

#### T cells

Expression of periostin is induced in epithelial cells in response to inflammatory cytokines, including signature cytokines of type 2 immune responses, such as IL-4 and IL-13 [16, 27]. Expression of periostin is also induced in lung fibroblasts by the anti-inflammatory cytokine, TGF-β [16, 27]. Some studies have shown that periostin-deficient mice have increased serum levels of IgE and peripheral Th2 responses, and also exhibit airway resistance, mucus production, and decreased production of lung TGF-β [43]. However, decreased mucous metaplasia and lower levels of IL-4 expression have also been reported. The mechanism by which periostin negatively affects allergen-induced responses may involve augmentation of TGF-β-induced T regulatory cell differentiation [17]. Correspondingly, a protective role for periostin and TGF-β has been demonstrated in IgE-mediated allergies and airway hyperresponsiveness [17]. Serum levels of periostin have also been found to reflect local production levels of periostin in inflamed lesions that are induced by Th2-type immune responses, and these levels have been used to predict the efficacy of Th2 antagonists against bronchial asthma [4].

### Periostin in experimental models of asthma

The murine model of asthma treated with HDMs has been extensively studied. In this model, *Postn*^+/+^ mice have exhibited increased airway responsiveness [67], as well as elevated levels of periostin expression in the airways, not only in the peribronchial inflammatory cells, but in the fluid lining of the airways as well [59]. *Postn*^+/+^ and wild-type mice have also exhibited significantly elevated levels of allergic airway inflammation, with an increased number of eosinophils present compared with periostin-deficient mice [5, 12, 26, 43]. In contrast, *Postn*^−/−^ mice have exhibited higher goblet cell metaplasia, higher serum IgG E levels, and increased peripheral Th2 responses compared to wild-type mice [16]. In addition, compared with wild-type controls, periostin-deficient mice develop increased AHR and serum IgE levels following allergen challenge, while mucus metaplasia and peribronchial fibrosis remain unaffected [17]. *Postn*^+/+^ mice also manifest significantly higher airway responsiveness to HDMs compared to *Postn*^−/−^ mice, and the transfer of bone marrow-derived DCs from *Postn*^+/+^ mice is sufficient to promote allergic responses in HDM-exposed *Postn*^−/−^ mice [26]. In a murine model of eosinophilic rhinosinusitis with nasal polyps to OVA, *Postn*^−/−^ mice exhibited enhanced polyp-like lesion formation and mast cell infiltration [65]. Furthermore, in a murine model of allergic airway inflammation, the sensitization and challenge of periostin-deficient mice with OVA resulted in an increased peripheral Th2 response compared with control mice [16].

### Periostin in human asthma

The inflammatory response in asthma is heterogeneous and involves many types of cells and cellular elements. It is important to recognize the different inflammatory phenotypes of asthma in order to understand the underlying disease processes. The different inflammatory phenotypes are also clinically relevant based on their potential to differentiate responses to therapeutic interventions [28]. Simpson et al. have classified asthmatic subjects into four groups based on the presence of neutrophils and eosinophils, with the 95^th^ percentile from healthy control subjects used as a cut-off point [68]. The four inflammatory subtypes included: neutrophilic asthma, eosinophilic asthma, mixed granulocytic asthma, and paucigranulocytic asthma. In recent years, many studies have elucidated the distinct mechanisms of these subgroups, and these have corresponded with the differential responses to therapy that have been observed. The mechanisms of eosinophilic asthma involve activation of Th2 pathways, typically by an allergen, and the release of Th2 cytokines, such as IL-4, −5, −9 and −13. Bronchial biopsies from these patients have shown an infiltration of eosinophils, activated mast cells, and T cells that are predominantly Th2 cells [28].

It is hypothesized that periostin plays an important role in eosinophilic forms of asthma [31]. In murine models, periostin has been linked to more severe asthmatic airway inflammation responses and hyperresponsiveness [26]. In humans, periostin has been found to prolong Th2/eosinophilic inflammation and to aggravate airway remodeling [69, 70]. These effects have also been associated with up-regulation of IL-4/13 [26, 27, 37, 66], and an increased number of eosinophils in the airways [47, 71, 72]. Furthermore, levels of periostin reflect persistent eosinophilic airway inflammation in severe asthmatics despite a high dose of ICS [31]. The latter can be explained in the context of an inflammatory phenotype which involves an increase in levels of Th2 cytokines, increased asthma severity, atopy, late-onset disease, and steroid refractoriness [19]. To date, cases of human asthma have not been analyzed for levels of periostin according to inflammatory phenotype. Furthermore, the available data have only demonstrated that expression of periostin is higher at both the gene and protein levels in asthmatic serum and sputum samples [31, 47, 51, 69–71, 73]. More recently, however, a greater role for periostin has been identified in relation to fibroblasts and airway epithelial cells in models of asthma (Fig. 2), while fewer studies have addressed the relationship between periostin and other inflammatory cells. Therefore, it will be important to elucidate the role of periostin in the mechanisms that mediate the various asthma phenotypes.

## Conclusion

Periostin is a multifunctional protein that is expressed by a variety of inflammatory cells. In addition, over expression of periostin has been observed in various types of disease concomitant with an increase in the number of inflammatory cells present. Periostin was first characterized as a matricellular protein, and since has been well-characterized as an important regulator of inflammatory cell infiltration and activation. In the field of asthma research, the role of periostin in relation to airway epithelial cells and fibroblasts has been of great interest, as well as its role in the pathogenesis of asthma caused by subcutaneous fibrosis. This perplexing paradox may be explained by considering the heterogeneity of airway inflammation in asthma and the specific effects of periostin in mediating eosinophilic forms of asthma. The role of macrophage also remains unclear, especially in relation to asthmatic inflammation. Furthermore, the cell types that mediate various asthmatic conditions are an important consideration. To date, there are limited data available regarding the levels and function of periostin in human asthma and in chronic obstructive pulmonary disease. Therefore, targeting the actions of periostin may help elucidate the underlying molecular mechanisms of asthma and may represent a promising strategy for the development of effective therapeutic agents for the treatment of asthma.

## Abbreviations

BAL: Bronchoalveolar lavage
TGF: Transforming growth factor
ECM: Extracellular matrix
IIPs: Idiopathic interstitial pneumonias
IPF: Idiopathic pulmonary fibrosis
AHR: Airway hyperresponsiveness
HDM: House dust mite
AD: Atopic dermatitis
PV: Psoriasis vulgaris
TARC: Thymus and activation-regulated chemokine
LDH: Lactate dehydrogenase
OVA: ovalbumin
FLSs: Fibroblast-like synoviocytes
RA-FLSs: Rheumatoid arthritis patients
IL: Interleukin
BLM: Bleomycin
GM-CSF: Granulocyte-macrophage colony-stimulating factor
mAb: Monoclonal antibody
Ecs: Endothelial cells
ERK1/2: Extra cellular signal-regulated kinases 1 and 2
PI3K: Phosphatidylinositol 3-kinase
DCs: Dendritic cells
GCM: Goblet cell metaplasia
SCF: Stem cell factor
